# Predatory Potential of Nymphal Odonates on *Aedes aegypti* Developing in Freshwater and Brackish Water Habitats

**DOI:** 10.3390/insects15070547

**Published:** 2024-07-19

**Authors:** Sivasingham Arthiyan, Thampoe Eswaramohan, Andrew Hemphill, Sinnathamby Noble Surendran

**Affiliations:** 1 Centre for Research in Entomology (CRE), Department of Zoology, Faculty of Science, University of Jaffna, Jaffna 40000, Sri Lanka; eswaramohan@univ.jfn.ac.lk (T.E.); noble@univ.jfn.ac.lk (S.N.S.); 2 Institute of Parasitology, Vetsuisse Faculty, University of Bern, 3012 Bern, Switzerland; andrew.hemphill@unibe.ch

**Keywords:** biological control, clearance rate of predator, dengue vector control, predatory rate, Sri Lanka

## Abstract

**Simple Summary:**

*Aedes aegypti*, the primary vector of dengue, undergoes preimaginal development in freshwater and brackish water habitats in northern Sri Lanka and other parts of the world. Dengue vector control in the form of source reduction targets only freshwater habitats. Since biological control is a component of integrated vector control, the present study investigated the predatory efficacy of dragonfly nymphs and damselfly nymphs that develop in both freshwater and brackish water habitats targeting *Ae. aegypti* larval forms in laboratory conditions. The dragonfly nymph *Hydrobasileus croceus* and the damselfly nymph *Paracercion hieroglyphicum* were found to be the most effective among six different species tested in both freshwater and brackish water conditions. The findings suggest that these odonate nymphs could serve as biological control agents to target preimaginal forms of dengue vectors under varying salinity conditions.

**Abstract:**

*Aedes aegypti*, the primary vector of dengue, undergoes preimaginal development in brackish water (BW). However, dengue vector control exclusively targets freshwater (FW) habitats. The present study evaluated the predatory efficacy of nymphal odonates that can develop in both FW and BW. Nymphs of three damselfly and three dragonfly species from FW and BW habitats were identified and acclimatized to FW (<0.5 gL^−1^ salt) and BW (10 gL^−1^ salt) mesocosm conditions. The experiment was repeated nine times with nine different individual predators per species under both salinity conditions. One hundred L3 *Ae. aegypti* from FW and BW laboratory colonies were introduced to determine the predatory rate (PR) and clearance rate (CR) after 24, 48, and 72 h, and one hundred L3 larvae were introduced every 24 h. The dragonfly nymph *Hydrobasileus croceus* and the damselfly nymph *Paracercion hieroglyphicum* showed the highest PR and CR under both rearing conditions at all times. However, damselfly and dragonfly nymphs significantly (*p* < 0.05) differed in their CR under both FW and BW conditions. Thus, all six odonate species have predatory potential and this suggests that they could be used as biological control agents to eliminate preimaginal stages of *Ae. aegypti* developing in both FW and BW habitats.

## 1. Introduction

Dengue is a viral disease that is transmitted by *Aedes* mosquitoes, causing up to 400 million infections. Approximately 100 million people acquire the disease, of which 40,000 die from severe dengue, most notably in tropical and subtropical nations. Almost half of the world’s population lives in areas with a risk of dengue [1]. Sri Lanka as a whole, and the Jaffna district in the north particularly, have been endemic for dengue inflicting high morbidity for decades [1,2]. A total of 80,106 and 4124 dengue cases were reported for the year 2023 for the whole country and the Jaffna district, respectively [3]. The mosquito species *Aedes aegypti* and *Ae. albopictus* are considered primary and secondary dengue vectors, respectively [4].

Mosquitoes require aquatic environments for the development of their preimaginal forms [5]. Over many years, dengue mosquitoes have thrived and adapted to various freshwater (FW) habitats including rainwater pools, leaf axils, water storage tanks, and discarded containers [1,6]. This adaptation extends across suburban and urban areas [2,7]. *Aedes aegypti* and *Ae. albopictus* have been considered obligate FW mosquitoes and thus vector control measures, such as eliminating preimaginal development habitats and applying larvicides, are designed to target exclusively FW habitats [4,8].

**Figure 1:**
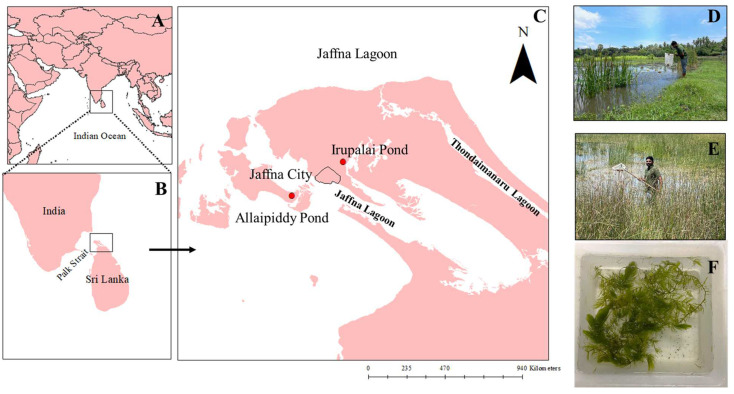
Geographical maps showing (**A**) the location of Sri Lanka on the Indian subcontinent, (**B**) the location of Jaffna, Sri Lanka, in the Indian ocean, and (**C**) the Jaffna district with major lagoons in northern Sri Lanka. Odonates were collected at the Jaffna city limits, and ponds where the sampling of predators was undertaken are marked as round solid red points. (**D**,**E**) show sampling at Irupalai and Allaipiddy ponds, respectively. (**F**) shows the experimental mesocosm set up.

*Aedes aegypti* and *Ae. albopictus* have been initially shown to undergo natural preimaginal development in FW as well as BW habitats in the Jaffna peninsula of northern Sri Lanka (Figure 1) [9]. FW, BW, and saline water contain < 0.5, 0.5–30, and >30 g/L of salt, respectively [9]. Since the Jaffna Peninsula has an extensive coastal line with many seawater inlets, there is a possibility for sea water intrusion into the available FW aquifers, causing rapid ground water salinization, and ultimately leading to the development of *Aedes* in BW [2,6]. It has been postulated that global climate change acts synergistically with the elevation of the sea level and will eventually expand coastal areas in many countries [10]. This is expected to provide suitable habitats for BW developing dengue vectors to transmit dengue in coastal areas.

In the absence of a licensed vaccine, vector control remains the main option to reduce dengue transmission [1,11]. Biological control is a component of an integrated vector control strategy [12]. Various studies have reported the effectiveness of using odonates such as the dragonfly *Bradinopyga geminata* to control and reduce mosquito populations [13]. Previous studies have shown varying predation rates by different odonate species on mosquito larvae under laboratory conditions [14] with different water volumes [15]. With this background, the present study was carried out to assess the efficacy of natural odonate predators from both FW and BW habitats in controlling *Ae. aegypti* populations.

## 2. Materials and Methods

### 2.1. Ethical Statement

The study received ethical approval for rearing mosquitoes and experimental insects from the University of Jaffna Animal Ethical Review Committee [AERC/2020/01(v2) and AERC/2023/03(v3)].

### 2.2. Freshwater and Brackish Water Aedes aegypti Colonies

Third instar larvae (L3) of *Ae. aegypti* were obtained from well-established laboratory colonies maintained under FW (≤0.5 g/L salt) and BW (10 g/L salt) conditions (27 ± 2 °C temperature and 75 ± 5% humidity) [16]. To maintain *Aedes aegypti* colonies, larvae were provided with powdered fish meal twice a day, and the adult females were fed on Balb/c mouse every three days, and 10% glucose pledgets at other times [16] at the Medical Entomology Laboratory of the Department of Zoology, Faculty of Science, University of Jaffna.

### 2.3. Field Survey to Identify the Presence of Odonate Nymphs in Potential Aedes Larval Development Habitats

A field survey was carried out from May 2022 to October 2023 within the Jaffna city limits (Figure 1C) to identify odonate nymphs present in water habitats, such as wells, earthen tanks, discarded pots, discarded large containers, earthen tanks, and overhead tanks, which are considered to be potential *Aedes* development sites in Jaffna city [2]. Weekly visits were made to collect odonate nymphs from the habitats using aquatic dip nets and standard aquatic dippers (Figure 1B,D,E) [17]. Collected samples were brought to the laboratory for species identification.

### 2.4. Species Identification of the Nymphal Predators

All the predatory insects used in the experiment were identified up to the species level using standard taxonomic keys [18,19,20,21]. The species level identification was then further confirmed using DNA sequence analysis. DNA was extracted from individual nymphal predators which were morphologically identified, as described previously [22,23]. The extracted DNA was subjected to polymerase chain reaction (PCR) amplification by targeting a segment of the mitochondrial cytochrome c oxidase subunit I (*COI*) gene using the primer pair LCO1490 (Forward-5′-GGT CAA CAAATC ATA AAG ATATTG G-3′) and HCO2198 (Reverse-5′-TAA ACTT CAG GGT GAC CAA AAA ATC A-3′) [24]. Each 25 μL PCR reaction contained GoTaq^®^ Green Master Mix (Promega, Madison, WI, USA), 2 mM MgCl2, 1 μL (100 pmol/μL) of each primer, and 5 μL of DNA. The samples were initially heated at 94 °C for 5 min, followed by 30 cycles of amplification at 94 °C for 30 s, 45 °C for 30 s, and 72 °C for 30 s, with a final extension at 72 °C for 7 min [25]. PCR products were purified using the QIAquick^®^ PCR Purification Kit (QIAGEN, Hilden, Germany) as per the manufacturer’s instructions, and the purified PCR products were sequenced bidirectionally at Macrogen Inc., Seoul, South Korea, Republic of Korea. The DNA sequences were edited in Finch TV (Geospiza Inc, Washinton, DC, USA) and aligned in ClustalW in MEGA 6.0 software [26]. The resultant sequences were compared with available sequences in the database of the National Center for Biotechnology Information (NCBI BLASTn search) to confirm morphology-based identification based on >98% similarity. The DNA sequences were deposited in GenBank under the accession numbers indicated in Table 1.

### 2.5. Collection and Maintenance of Odonate Nymphal Predators for the Experiment

Immature stages of predatory odonates were collected from two natural ponds (Figure 1) located at Irupalai (9°41′6″ N 80°03′46″ E, Figure 1D) and Allaipiddy (9°37′29″ N 79°58′20″ E, Figure 1E). The collected nymphs were reared in the insectary of the Department of Zoology, University of Jaffna. The water salinity of both ponds was measured using a handheld refracto-salinometer (Atago Co. Ltd., Tokyo, Japan). The predators found to be common for both ponds were chosen for subsequent predatory experiments. The collected nymphal predators were acclimatized for a week to FW (0.5 g/L salt) and BW (10 g/L salt) conditions. The FW condition for the experiment was obtained by mixing the tap water and the pond water from Irupalai in equal amounts. Similarly, seawater, pond water from Allaipiddy, and tap water were mixed to obtain BW. Nymphal predators collected from the FW pond (Irupalai) and BW pond (Allaipiddy) were used for the FW and BW experiments, respectively. The pond water, with the naturally available biota such as protozoans and planktonic algae [17,27], was used without any mosquito larvae in order to avoid prey familiarization and learning [28].

The total length of the nymphs was measured with a vernier caliper (XIGONG, Wuxi, Jiangsu, China), rounded to the nearest millimeter, and nymphs within the size range of 1.2–1.8 cm were selected for the experiment as nymphs within the size range of 1–2 cm were reported as late instar [17,28]. Prior to the commencement of the experiment, the predators were subjected to starvation for 24 h [28,29].

### 2.6. Evaluation of the Predatory Efficiency of Different Odonates Using Mesocosm Set-Ups

Predatory experiments were conducted in mesocosm set-ups (semi natural habitats to mimic the natural environment) [30] to assess the efficiency of predation by nymphal predators as described previously [30,31,32,33] with slight modifications, and the experiments were performed nine times. The predators were placed in separate holding tanks with the available aquatic plants from the natural ponds, each measuring 30 cm × 27 cm × 12 cm and containing 5 L of either FW or BW (Figure 1F). The tanks were covered with mesh screening on top and maintained under standard environmental conditions of 27 ± 2 °C temperature and 75 ± 5% humidity with direct solar illumination through windows.

In each holding tank, 100 L3 *Ae. aegypti* and a single predator were introduced [17], and the number of surviving mosquito larvae was recorded every 24 h for a total of 72 h [33,34]. Control experiments were set up for both salinity conditions, where 100 L3 *Ae. aegypti* were introduced without nymphal predators with the ad libitum supply of fish pellets. To maintain a constant larval density of 100 individuals per tank, remaining larvae were counted and removed and then new batches of 100 L3 larvae were added to the tanks at 24 h intervals [17].

### 2.7. Data Collection and Statistical Analysis

Predatory rate (PR), a measure of percentage of the prey killed or eaten by the predator, was calculated as the percentage of number of consumed larvae, which was calculated by subtracting the remaining larvae from the initial 100 larvae at each time point [35]. The clearance rate (CR) is the measure of the percentage of prey killed per day per predator [31] and the predatory impact (PI) is the killing ability of the prey [32]. The predatory rate, clearance rate, and predatory impact were determined as follows;

Predatory rate (PR) = [(100 − Existing larva)/100] × 100%

Clearance rate (CR) = [V × (ln P)]/[T × N]

CR = Clearance rate of a predator (% of prey killed in liters/day/predator);

V = Volume of water in liters;

P = % of prey killed or eaten;

T = Time (in days);

N = No. of predators;

Predatory impact (PI) = Number of larvae consumed by a predator per hour.

The significant association between the clearance rate and the predatory impact of the predators for third instar larval *Ae. aegypti* considering species, salinity, and the predatory hours were calculated using the non-parametric Kruskal–Wallis test, while the significant difference on CR between the freshwater and brackish water conditions was calculated using non-parametric Mann–Whitney U-Test using R software [Version 4.2.1, R Core Team (2022)] as the data were not normally distributed. Similarity-based clustering of the predators was done with the Analysis of Similarities (ANOSIM) using the same software.

## 3. Results

### 3.1. Field Collection of Nymphal Odonates from Aedes Developing Habitats

A total of 59 different mosquito developing habitats were sampled during the survey, including 12 wells, 4 discarded pots, 8 discarded large containers, 16 earthen tanks, and 19 overhead tanks. Dragonfly nymphs were present in seven habitats representing two wells, two discarded pots, two overhead tanks and an earthen pond. All of the nine collected dragonfly nymphs were identified as *Pantala flavescens* developing in FW habitats. This is the first report documenting the presence of the nymphal dragonfly *P. flavescens* in the earthen tanks, overhead tanks, and discarded pots in domestic areas in Jaffna city (Figure 1C).

### 3.2. Field Collection of Nymphal Odonates from the Ponds

The average salinity of both ponds, Irupalai pond (Figure 1D) and Allaipiddy pond (Figure 1E), was ≤0.5 g/L salt and 6 g/L salt, respectively. A total of 39 dragonfly nymphs and 37 damselfly nymphs were collected from the FW Irupalai Pond, whereas 37 dragonfly nymphs and 41 damselfly nymphs were collected from the BW Allaipiddy Pond. This is the first observation of FW nymphs present in BW habitats. Among the nymphal dragonflies, *Hydrobasileus croceus* was the longest (16.1 ± 0.9 mm; mean ± standard deviation) and *Brachydiplax sobrina* was the shortest (15.8 ± 1.0 mm). Among the nymphal damselflies *Ceriagrion coromandelianum* (16.5 ± 0.7 mm) and *Paracercion v-nigrum* (16.3 ± 0.8 mm) were the longest and the shortest, respectively (Table 1).

### 3.3. Species Identification and Their Confirmation

Six different nymphal predator species were used for the present study: the dragonfly nymphs *Pantala flavescens*, *Hydrobasileus croceus,* and *Brachydiplax sobrina* and the damselfly nymphs *Ceriagrion coromandelianum*, *Paracercion v-nigrum,* and *Paracercion hieroglyphicum*. All of the predators were found in both FW and BW ponds. The details of the predators used in the experiment are tabulated along with the GenBank accession numbers and the similarity percentage of the species obtained from the BLASTn search (Table 1).

### 3.4. The Predatory Rate (PR) and Predatory Impact (PI) of the Predators in Freshwater and Brackish Water Experiments

On average, the predatory rate (percentage of prey consumed by a predator per day) of the tested predators was above 50% for all of the species with one exception: the damselfly nymph *C. coromandelianum*, which had ≤45% for both FW and BW experimental conditions at 24 and 48 h, but showed a 57 ± 17 and 57 ± 14 (mean% ± sd) predatory rate under FW and BW experimental conditions, respectively, after 72 h (Table 2). In general, the predatory rate of the predators increased with time for both FW and BW, except for the damselfly nymph *P. hieroglyphicum*, for which the predatory rate declined after 24 h (Table 2) in BW. However, the predatory rate of the damselfly nymph *C. coromandelianum* increased with time in BW when compared with FW (Table 2).

The predatory impact is an indirect indicator of the predatory rate documenting the consuming ability of the predators. The predatory impact value indicates the number of prey consumed by a predator per hour [32]. Among all of the predators, *H. croceus* had the highest predatory impact of four larvae per hour for both FW and BW conditions, while *C*. *coromandelianum* has the lowest predatory impact of two and one larva per hour for FW and BW conditions, respectively (Table 2).

### 3.5. Clearance Rate of the Predators in Freshwater and Brackish Water Experiments

The results of the *Ae. aegypti* larval clearance rate by experimental nymphs are summarized in Table 3. The clearance rate was measured as the percentage of prey killed per day per predator in five liters of water [31]. Among the three dragonfly species, *H. croceus* had the highest clearance rate of 22.7 ± 0.1 (mean ± standard deviation) after 24 h and 48 h, and a clearance rate of 22.8 ± 0.1 after 72 h in FW conditions, and 22.8 ± 0.1, 22.7 ± 0.2, and 22.7 ± 0.1 after 24, 48, and 72 h, respectively, in BW conditions. Among the damselflies, *P. hieroglyphicum* had the highest clearance rate at 20.0 ± 2.7, 20.2 ± 1.9, and 21.0 ± 2.3 in FW for 24 h, 48 h, and 72 h, respectively, and 22.0 ± 0.5, 21.2 ± 1.6, and 21.6 ± 0.4 in BW for 24 h, 48 h, and 72 h, respectively. Among the three dragonfly nymphs, *H. croceus* showed (*p* < 0.05) the highest clearance rate in both FW and BW conditions at all the time points (*p* < 0.05). Among the damselfly nymphs, *C. coromandelianum* showed the lowest clearance rate in both FW and BW only at the 48 h time point (*p* < 0.05). Although the damselfly nymph *P. hieroglyphicum* had the highest predatory rate, it did not show significant *(p* > 0.05) difference on the clearance rate with the damselfly *P. v-nigrum* at all of the time intervals for FW and BW conditions. The study also reveals that there is a significant (*p* < 0.05) difference in clearance rate between damselfly and dragonfly nymphs reared in FW and BW conditions (Table 3). However, there is no significant (*p* > 0.05) difference present for the clearance rate among the species under both rearing conditions at all of the time points (Table 3). Therefore, all of the predators could be used in FW and BW habitats for the control of the immature forms of *Ae. aegypti*.

### 3.6. Cluster Analysis on the Clearance Rate (CR) of the Nymphal Predators

**Figure 2:**
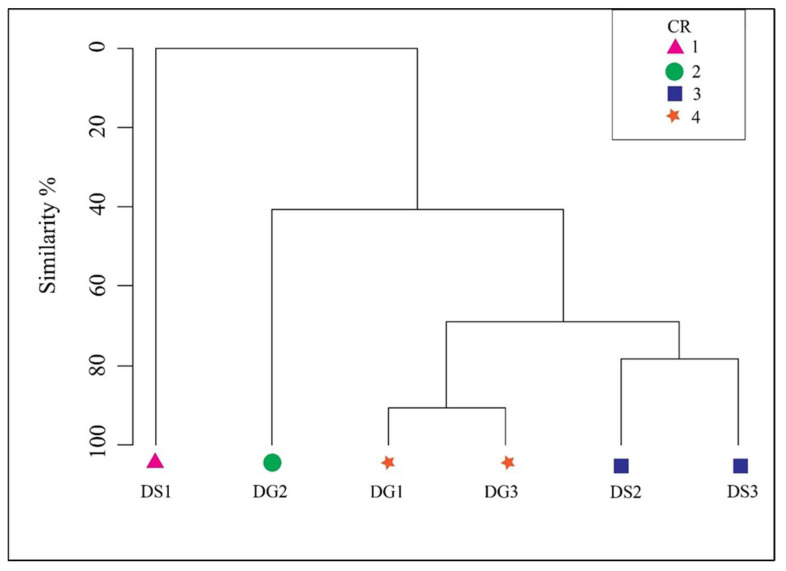
Dendrogram of the cluster analysis of the odonates in terms of the *Aedes* larval consumption patterns, where the numbers from 1–4 represent the different clusters and organisms represent the clusters DG—dragonfly and DS—damselfly. DG1: *Pantala flavescens*, DG2: *Hydrobasileus croceus*, DG3: *Brachydiplax sobrina*, DS1: *Ceriagrion coromandelianum*, DS2: *Paracercion hieroglyphicum*, and DS3: *Paracercion v-nigrum*.

The dendrogram resulting from cluster analysis using the Bray–Curtis similarity index indicated the formation of four distinct clusters among the predatory odonates (Figure 2). Specifically, the damselfly *C. coromandelianum* and the dragonfly *H. croceus* constituted the first and second clusters, respectively. The species *P. hieroglyphicum* and *P. v-nigrum* formed the third cluster, with a similarity of 77% between them. The fourth cluster was comprised of *P. flavescens* and *B. sobrina*, which shared a similarity of 90% based on the average CR (24–72 h) of the odonate nymphs preying on L3 *Ae. aegypti* (Figure 2). Furthermore, the third cluster (damselflies) and the fourth cluster (dragonflies) demonstrated a 69% similarity in their CR. The clustering status of the dendrogram (Figure 2) was statistically confirmed to be statistically significant (*p* < 0.05) from the Analysis of Similarities (ANOSIM).

## 4. Discussion

The present study reveals that the dragonfly and damselfly species assessed in this experiment develop naturally in FW and BW habitats and could be used as biological control agents to reduce dengue vector populations. This is the first report that describes the potential predatory application of dragonfly nymphs and damselfly nymphs to reduce *Aedes* preimaginal forms that develop in FW and BW habitats. The predatory potential can be expressed in terms of PI [32], predatory rate, and clearance rate [31,36], where predatory rate is the actual percentage of the prey killed or eaten by the predators, the PI is the prey killing ability of the predators, and the clearance rate stands for the combined effect of searching ability, killing, and consumption by the predators and prey evasion in unit time and space [34]. Therefore, the clearance rate is considered an effective measure to determine predatory efficacy in a mesocosm set up as discussed in this study. This study shows that the studied dragonflies have a higher clearance rate compared to the damselflies investigated, suggesting that nymphal dragonflies could represent a preferred choice as biological control agents.

The dragonfly *H. croceus* exhibited the highest clearance rate among all odonate predators for L3 *Ae. aegypti* in both FW and BW experiments at all time points. The damselfly nymph *C. coromandelianum* exhibited the lowest clearance rate initially but showed an increasing trend over the time regardless of water salinity. This suggests that *C. coromandelianum* becomes an effective predator with prey familiarization.

The cluster analysis revealed a meaningful explanation based on the clearance rate of tested odonates. The first and the second clusters representing the damselfly *C. coromandelianum* and dragonfly *H. croceus,* respectively, indicate that the first clustering reflected the lowest clearance rate, and the second cluster was due to the highest clearance rate. The damselfly *C. coromandelianum* has no (0%) similarity with other species due to its lowest clearance rate. Though the dragonfly *H. croceus* showed maximum predation in this study, it shares only 40% similarity with the other four predators. This suggests that *H. croceus* can be considered a highly potent predator among the six odonates that were assessed, to be released in the field as biological control agent in FW and BW habitats.

The nymphal stage of dragonflies typically undergoes an average of 11–14 molts, with an actual range spanning from 10 to 20 molts. This development process can vary significantly across species, with some Epiophlebiidae species, for instance, requiring anywhere from three months to an exceptional duration of 6–10 years to complete their nymphal development [21]. Such a prolonged developmental span of these predators will be an additional advantage with respect to mosquito control throughout the year.

Biological control of mosquitoes encompasses a variety of organisms, notably larvivorous fishes such as *Gambusia* and *Poecilia* [27,37]. Successful implementation of copepods for dengue vector control has been documented in Vietnam [38], although copepods are constrained in their habitat range [39]. Tadpoles are reported to consume *Aedes* sp. eggs [40]. Other insects such as hemipterans, coleopterans, and odonates (particularly dragonflies), have been extensively studied for their predatory efficacy on mosquito immature forms in both field and laboratory settings [30,34,41,42,43]. Research from Kerala, India, highlighted the dragonfly *Bradinopyga geminata* as the most effective predator of *Ae. aegypti* larvae based on PI [34].

A previous study from Sri Lanka evaluated the predatory efficacy of five dragonfly nymphs against *Ae. aegypti* (L4) under FW conditions [17]. The present study tested five new odonate species along with *P. flavescens,* which was included in the previous study [17]. However, there is no report on the predation of the nymphal damselflies from Sri Lanka to date. Notably, the dragonfly *P. flavescens* and the damselfly *C. coromandelianum* were only tested in other regions for predatory efficacy among the tested odonates from the present study; the predatory efficacy of the dragonfly *P. flavescens* was evaluated in India [42] and Pakistan [43] against *Aedes* and *Culex* species, respectively, and the predatory efficiency of the damselfly *C. coromandelianum* was assessed in India [33] against *Ae. aegypti* under FW condition.

The development of dragonflies in small water bodies in peri domestic areas in Sri Lanka has not been reported previously. The field observations in Jaffna city suggest that the predator dragonfly nymph *P. flavescens* is adapted to develop in water habitats such as wells, earthen tanks, overhead tanks, and pots where dengue vectors undergo preimaginal development. Therefore, *P. flavescens* can be considered one of the potential candidates to control dengue vectors in peridomestic areas under rural and urban settings in Sri Lanka where water habitats are either FW or BW.

Most biological control studies using odonates have been conducted under laboratory conditions. Designing a field-based experiment with odonates demands mass rearing, which is particularly challenging in a laboratory setting [27]. This is especially true for dragonflies, due to their long lifespan, variations in nymphal instars, and tendencies towards cannibalism [44]. Conversely, rearing damselflies in the laboratory has proven feasible, as they are smaller, have shorter generation times, and can reproduce continuously without hibernation [44].

Despite the above challenges, a field study was conducted in Japan to suppress the *Ae. albopictus* larval population by introducing the laboratory-reared dragonfly nymphs *Sympetrum frequens* and *S. infuscatum* into the ovitraps. The study concluded that the dragonfly nymphs were effective in reducing larval *Aedes* populations [45]. Moreover, facilitating the predators to continue their lifecycle in habitats such as cement tanks and artificial containers, where dengue vectors undergo preimaginal development, could accelerate the reduction of vector populations. This was demonstrated in a field experiment in Germany, where the populations of *Aedes* and *Culex* mosquitoes were effectively controlled by the dragonfly *Bradinopyga strachni*, which colonized 35 concrete containers [46].

## 5. Conclusions

The present study reveals that the dragonfly nymph *H. croceus* is an effective predator of larvae of the primary dengue vector *Ae. aegypti* which naturally undergoes preimaginal development in both freshwater and brackish water habitats. Furthermore, the dragonfly *P. flavescens* could also be released in potential dengue vector developing habitats in peridomestic areas of urban and rural environments contributing to mosquito control. However, these laboratory findings require validation through field experiments in the future.

## Figures and Tables

**Table 1:** Details of the predator species used in the experiment. The scale bar represents 1 cm.

Predator Name	Mean Predator Length in mm (Range)	NCBI Accession No./Similarity Percentage (BLASTn)	Image
***Pantala flavescens*** **Order: Odonata** **Infraorder: Anisoptera** **Family: Libellulidae**	16.0 ± 1.2 (sd) (14.8–17.2)	OR342309/99%	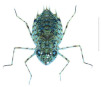
***Hydrobasileus croceus*** **Order: Odonata** **Infraorder: Anisoptera** **Family: Libellulidae**	16.1 ± 0.9 (15.2–17.0)	OR336051/99%	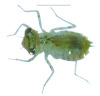
***Brachydiplax sobrina*** **Order: Odonata** **Infraorder: Anisoptera** **Family: Libellulidae**	15.8 ± 1.0 (14.8–16.8)	OR336316/99%	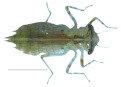
***Ceriagrion coromandelianum*** **Order: Odonata** **Suborder: Zygoptera** **Family: Coenagrionidae**	16.5 ± 0.7 (15.8–17.2)	OR362797/99%	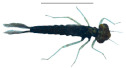
***Paracercion hieroglyphicum*** **Order: Odonata** **Suborder: Zygoptera** **Family: Coenagrionidae**	16.5 ± 0.6 (15.9–17.1)	OR512024/99%	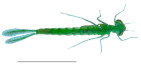
***Paracercion v-nigrum*** **Order: Odonata** **Suborder: Zygoptera** **Family: Coenagrionidae**	16.3 ± 0.8 (15.5–17.1)	OR512029/98%	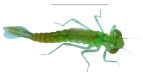

**Table 2:** The predatory rate (PR) and predatory impact (PI) of the predators at different time points under FW and BW conditions.

Common Name	Species Name	24 h				48 h				72 h			
		FW		BW		FW		BW		FW		BW	
		PR	PI	PR	PI	PR	PI	PR	PI	PR	PI	PR	PI
Dragonfly	*Pantala flavescens*	68 ± 23	3 ± 1	65 ± 24	3 ± 1	85 ± 8	4	78 ± 20	3 ± 1	89 ± 8	4	84 ± 18	3 ± 1
	*Hydrobasileus croceus*	94 ± 2	4	95 ± 2	4	94 ± 2	4	93 ± 4	4	95 ± 2	4	94 ± 3	4
	*Brachydiplax sobrina*	68 ± 23	3 ± 1	61 ± 21	3 ± 1	85 ± 8	4	74 ± 18	3 ± 1	89 ± 8	4	79 ± 15	3 ± 1
Damselfly	*Ceriagrion coromandelianum*	44 ± 23	2 ± 1	28 ± 12	1	41 ± 14	2 ± 1	45 ± 14	2 ± 1	57 ± 17	2 ± 1	57 ± 14	2 ± 1
	*Paracercion hieroglyphicum*	60 ± 23	3 ± 1	82 ± 8	3	60 ± 20	3 ± 1	72 ± 21	3 ± 1	72 ± 26	3 ± 1	76 ± 5	3
	*Paracercion v-nigrum*	58 ± 26	2 ± 1	55 ± 20	2 ± 1	64 ± 17	3 ± 1	65 ± 21	3 ± 1	61 ± 18	3 ± 1	65 ± 25	3± 1

h, hours; PR, Predatory Rate [percentage of larvae consumed per day (mean% ± sd)]; PI, Predatory Impact [number of larvae consumed per hour (mean ± sd)].

**Table 3:** Clearance rate (CR) of the predators at different time points under FW and BW conditions.

Common Name	Species Name	24 h			48 h			72 h		
		FW	BW	*p* Value	FW	BW	*p* Value	FW	BW	*p* Value
Dragonfly	*Pantala flavescens*	20.6 ± 3.0	20.5 ± 2.1	0.45	22.2 ± 0.5	21.6 ± 1.5	0.08	22.4 ± 0.5	22.0 ± 1.2	0.16
	*Hydrobasileus croceus*	22.7 ± 0.1	22.8 ± 0.1	0.85	22.7 ± 0.1	22.7 ± 0.2	0.29	22.8 ± 0.1	22.7 ± 0.1	0.53
	*Brachydiplax sobrina*	20.6 ± 3.0	20.2 ± 1.9	0.21	22.2 ± 0.5	21.4 ± 1.4	0.08	21.3 ± 2.4	21.9 ± 1.1	0.65
Damselfly	*Ceriagrion coromandelianum*	18.3 ± 2.7	16.2 ± 2.1	0.12	18.4 ± 1.6	18.9 ± 1.4	0.25	20.0 ± 1.6	20.1 ± 1.2	0.61
	*Paracercion hieroglyphicum*	20.0 ± 2.7	22.0 ± 0.5	0.07	20.2 ± 1.9	21.2 ± 1.6	0.12	21.0 ± 2.3	21.6 ± 0.4	0.38
	*Paracercion v-nigrum*	19.8 ± 2.6	19.8 ± 1.9	0.75	20.6 ± 1.3	20.6 ± 2.0	0.86	20.3 ± 1.7	20.5 ± 2.2	0.95

h, hours; percentage clearance rates (CR) with ± standard deviation (sd); *p* values were derived from the outcome of Mann–Whitney U-Test comparing CR of individual predators maintained in FW and BW.

## Data Availability

The data generated to support the findings of this study are included in the manuscript within the text.
